# MSNA during prolonged post-faint hypotension

**DOI:** 10.1007/s10286-012-0159-5

**Published:** 2012-03-14

**Authors:** J. Rozenberg, W. Wieling, I. K. Schon, B. Westerhof, C. Frampton, D. Jardine

**Affiliations:** 1 Department of Internal Medicine, Academic Medical Center, University of Amsterdam, Amsterdam, The Netherlands; 2 BMEYE, Academic Medical Center, Amsterdam, The Netherlands; 3 Department of Medicine, Christchurch Hospital, Christchurch, New Zealand; 4 De Clercqstraat 43-II, 1053 AC Amsterdam, The Netherlands

**Keywords:** Prolonged post faint hypotension (PPFH), Muscle sympathetic nerve activity (MSNA), Total peripheral resistance (TPR), Cardiac output (CO), Sympathetic withdrawal, Vagal outflow

## Abstract

**Background:**

Following tilt-induced syncope, blood pressure usually recovers rapidly after tilt back to the horizontal position. However, in some patients, hemodynamic recovery is delayed, a condition recently termed “prolonged post-faint hypotension” (PPFH). The mechanism is thought to be mediated by increased vagal outflow rather than exaggerated peripheral vasodilatation and sympathetic withdrawal. To date, no muscle sympathetic nerve activity (MSNA) recordings have been reported in this condition, so we aimed to confirm that neither vasodilatation nor MSNA withdrawal was responsible.

**Objectives:**

To retrospectively select patients with satisfactory recordings of continuous BP and MSNA during tilt-induced syncope. To compare hemodynamic and MSNA profiles in patients with PPFH to patients with normal recovery (NR) after tilt-back.

**Methods:**

All patients were studied in Christchurch, New Zealand, between 1998 and 2008 using continuous arterial BP monitoring, and microneurographic recordings of MSNA from the right leg. Only patients with satisfactory BP and MSNA data throughout baseline, head-up tilt and presyncope were selected. Stroke volume (SV), cardiac output (CO), and total peripheral resistance (TPR) were derived using Modelflow. After baseline measurements, patients were tilted to the head-up 60° position and given GTN spray if asymptomatic after 20 min. Following the onset of presyncope, patients were tilted slowly back to the horizontal. PPFH was defined as systolic BP <85 mmHg for at least 2 min after tilt-back. Measurements were averaged at baseline, early tilt, presyncope, early and late recovery. Within-group comparisons were made between baseline and all other time points. Between-group comparisons were made over all time points.

**Results:**

Patients with PPFH (7 males, age 46 ± 5 years, *n* = 8) and with NR (8 males, age 47 ± 6 years, *n* = 8) were selected. Presyncope was provoked by GTN in 4/8 patients in each group. In both groups, MAP remained below baseline during early and late recovery: PPFH 84 ± 5 versus 51 ± 5 and 64 ± 5 mmHg (*p* = 0.001, *p* = 0.001); NR 104 ± 5 versus 83 ± 5 and 93 ± 5 mmHg (*p* = 0.001, *p* = 0.03). However, MAP and HR were lower in the PPFH group (*p* = 0.004, *p* = 0.023). During early recovery, CO remained below baseline only in the PPFH group (*p* = 0.001), whereas TPR remained constant in both groups. In both groups, all MSNA indices tended to remain above baseline levels during early and late recovery. PPFH 25 ± 2 increased to 31 ± 6 and 29 ± 4 bursts/min (*p* = 0.09, 0.02); NR 23 ± 3 increased to 33 ± 3 and 34 ± 3 bursts/min (*p* = 0.06, 0.01).

**Conclusions:**

PPFH does not appear to be mediated by exaggerated vasodilatation or sympathetic withdrawal. Delayed recovery of cardiac output by increased vagal outflow is a more likely mechanism.

## Introduction

Until recently, the mechanism of vasovagal syncope was thought to be dual, vasodilatation mediated by sympathetic withdrawal and bradycardia mediated by vagal activation [1]. The concept of sudden reduction of sympathetic activity during faint causing relaxation of the blood vessels was based on plethysmographic demonstration of forearm vasodilatation [2–4], and microneurographic recordings showing withdrawal of muscle sympathetic nerve activity (MSNA) in the leg [5–9]. MSNA is considered to be a major determinant of rapid changes in total peripheral resistance (TPR) [10, 11]. Despite these important findings, other studies found that peripheral vasodilatation was not generalized and probably a terminal event in the time-course of the vasovagal reaction. Invasive studies [12–15] demonstrated that hypotension during presyncope was initiated by a fall in cardiac output which began up to 5 min before syncope. Continuous finger-pulse BP monitoring with Modelflow, confirmed that a gradual fall in cardiac output, secondary to stroke volume decay mediated progressive hypotension during presyncope [16]. This was consistent with the observation that presyncope could be rapidly reversed by maneuvers, which increased venous return and stroke volume [17, 18]. Therefore, decreasing cardiac output is clearly important in the sequence of hemodynamic changes before vasovagal syncope. Recently, Wieling et al. [19] described a new group of patients with a history of severe fainting. Continuous BP monitoring during tilt-induced syncope demonstrated prolonged post faint hypotension (PPFH) and the mechanism for this appeared to be mainly delayed recovery of cardiac output and reduced left ventricular contractility. TPR was maintained throughout the faint and so even in PPFH patients with severe fainting, no peripheral sympathetic withdrawal was found. So far, no MSNA measurements have been performed in PPFH patients. We hypothesized that because TPR was maintained during PPFH, MSNA would also remain increased from baseline values. We also hypothesized that PPFH patients might have lower resting TPR and MSNA levels compared to NR patients, predisposing them to more severe episodes of VVS.

## Methods

### Study population and protocol

We reviewed hemodynamic data from 340 recordings of tilt tests undertaken in the Christchurch Hospital Syncope Clinic from 1995 to 2005. All patients had a history of vasovagal symptoms over the 6 months before tilt. Of these, 203 tilt tests were positive. A positive tilt test was defined as a progressive fall in systolic blood pressure (SBP) below 100 mmHg associated with symptoms of impending syncope. PPFH, defined as SBP <85 mmHg for at least 2 min after tilt back was found in 25 patients (7.4% of tilt tests). From this group, on the basis of satisfactory MSNA recordings during tilt and presyncope (see below), we selected eight PPFH patients, (7 males, age 46 ± 5 years). Nearly all had mild bradycardia during late recovery (in 7 out of 8, HR fell below 60 bpm). However, only two of our PPFH patients had severe bradycardia (HR less than 50 bpm) as in the group described by Wieling et al. [19]. A further eight patients with satisfactory MSNA recordings and normal recovery (NR) of blood pressure after tilt-induced syncope were selected as controls (8 males, age 47 ± 6 years). Patients were included in the NR group provided SBP increased to >85 mmHg during the first 2 min of recovery.

Passive head-up tilt (HUT) to 60^o^ was performed on a hydraulic tilt-table between 9:00 AM and 1:00 PM in a temperature-controlled room (23°C) according to the Italian protocol [20]. If presyncope did not occur within 20 min, nitroglycerine (0.4 mg) was administered sublingually. When a progressive fall in blood pressure occurred, associated with prodromal symptoms, the patient was tilted slowly back to the horizontal over 20 s.

This study was approved by the Canterbury Hospital Ethics Committee.

### Data acquisition and analysis

Microneurography needles were positioned for recording MSNA from the right peroneal nerve [21]. The nerve was located behind the head of the fibula, and, with the use of transcutaneous stimulation, an insulated tungsten electrode with a 1- to 5-μm tip was inserted. The nerve signal was amplified (1,00,000×) filtered (700–2,000 Hz), integrated (time constant 0.1 ms), and displayed online with blood pressure and ECG. Bursts of sympathetic activity were indentified and counted each minute (bs/min) by the same operator (DLJ). The nerve signal was accepted if the following criteria were met: the signal-to-background ratio was greater than 3; the bursts were pulse-synchronous; burst amplitude was inversely proportional to diastolic BP; and skin activity was absent. It is difficult to maintain the recording field during recovery following the tilt-back procedure particularly when patients are severely hypotensive and symptomatic. Therefore, although we were able to maintain MSNA recordings throughout recovery in all of the selected NR patients, we only achieved this in 50% of the PPFH group.

Non-invasive beat-to-beat BP was measured at the finger (Finometer Blood Pressure Monitor) with the hand held at heart level in a sling. Invasive BP recordings were undertaken from a 3 French brachial arterial line with a deltrans transducer fixed at mid-atrial level. The measured signal was analogue to digital converted at 200 Hz, and stored on a hard disk for offline analysis. Systolic (SYS) and diastolic (DIAS) arterial pressures were derived from the arterial pressure waveform. Mean arterial pressure (MAP) was calculated from the integral of the arterial pressure wave over one beat divided by the corresponding beat interval. Heart rate (HR) was computed as the inverse of the inter-beat interval and expressed as beats per minute. Beat-to-beat changes in stroke volume (SV) were estimated by modeling flow from the arterial pressure waveform (Modelflow, TNO Biomedical Instrumentation), expressed in ml [22]. Cardiac output (CO), expressed in l/min, was the product of estimated SV and HR. Total Peripheral Resistance (TPR), expressed in mmHg s/ml, as Medical units (MU) was derived from MAP divided by the computed CO. The Modelflow method estimates aortic flow from finger arterial pressure by simulating a non-linear model of the aortic input impedance [23]. This pulse wave analysis method corrects for individual differences in age, gender, height and weight, permitting group average data to be examined accurately in subjects without structural or functional heart disease [24]. Previous head-up tilt experiments have demonstrated good agreement between Modelflow and traditional estimates of CO [25, 26].

### Sampling of time intervals

For each interval, hemodynamic variables were initially averaged over 30-s periods and then averaged (2 × 30 s periods for each minute) for statistical testing. MSNA variables were initially counted and measured over 60-s periods. Values for all variables are expressed as mean ± SE.

The following time intervals were analyzed:

1. Baseline: the last 5 min in the supine position before head-up tilt
2. Early HUT: the steady-state adjustment to orthostatic stress during the 1–3 min of HUT (60–180 s).
3. Presyncope: the last minute before tilt-back to the horizontal position.
4. Recovery: 0–2 and 4–5 min following tilt-back to the horizontal position.

### Statistical analysis

Statistical analysis was performed using unpaired *t* tests and χ^2^ tests for demography and baseline variables. Changes in variables with time were analyzed using one-way ANOVA for within-group and two-way ANOVA for between-group comparisons.

## Results (see Table 1 and Figs. 1, 2, 3)

All patients had satisfactory MSNA data during tilt and presyncope, but 4/8 in the PPFH group had incomplete data during recovery. The groups were matched for age and gender. Four patients with PPFH (and 2 with NR) had a life-long history of syncope associated with blood phobia (*p* = 0.6). Five PPFH patients reported protracted symptoms after fainting (feeling unwell for more than 6 h). Tilt time and requirement for GTN provocation were similar between groups.

**Table 1:** Patient characteristics and baseline hemodynamics

Variable	PPFH (*n* = 8)	NR (*n* = 8)	*p*
Age (years)	46 ± 5	47 ± 6	1.0
Gender (m)	7	8	1.0
Blood phobia	4	2	0.6
Syncopes per year	2 ± 0.7	2.8 ± 0.8	0.5
Tilt time (min)	18.5 ± 2	20.3 ± 3	0.6
GTN	4	4	1.0
MAP (mmHg)	84 ± 4	104 ± 5	0.01
HR (bpm)	59 ± 2	73 ± 6	0.07
SV (ml)	102 ± 8	77 ± 8	0.05
CO (l/min)	5.9 ± 0.5	5.5 ± 0.5	0.59
TPR (MU)	0.9 ± 0.1	1.4 ± 0.2	0.05
MSNA (bursts/min)	25 ± 2	23 ± 3	0.8
MSNA (bursts/100b)	43 ± 4	35 ± 7	0.2

Mean ± SE for demographics and baseline variables. All values quoted without units refer to absolute counts

*PPFH* prolonged post-faint hypotension, *NR* normal recovery, *GTN* glyceryl trinitrate given at 20 min head-up tilt, *tilt time* time tilted up before presyncope, *MAP* mean arterial pressure, *HR* heart rate, *SV* stroke volume, *CO* cardiac output, *TPR* total peripheral resistance, *MSNA* muscle sympathetic nerve activity

**Fig. 1:**
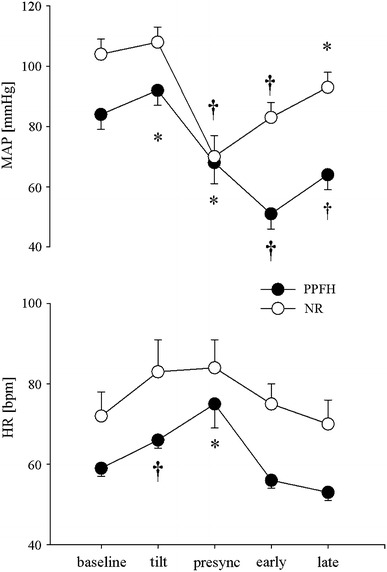
Mean and standard error for mean arterial pressure and heart rate at baseline, early tilt, presyncope, early and late recovery in patients with prolonged post-faint hypotension (PPFH *filled circles*) and patients with normal recovery (NR *open circles*). *Asterisks* and *dagger* refer to *p* < 0.05 and *p* < 0.01 for within-group comparisons to baseline

**Fig. 2:**
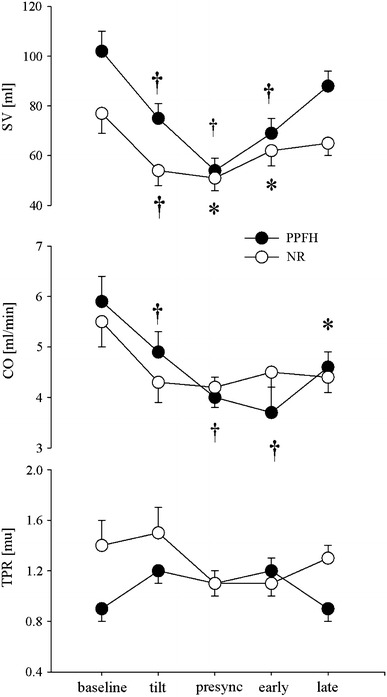
Mean and standard error for stroke volume, cardiac output and total peripheral resistance at baseline, early tilt, presyncope, early and late recovery in patients with prolonged post-faint hypotension (PPFH *filled circles*) and patients with normal recovery (NR *open circles*). *Asterisks* and *dagger* refer to *p* < 0.05 and *p* < 0.01 for within-group comparisons to baseline

**Fig. 3:**
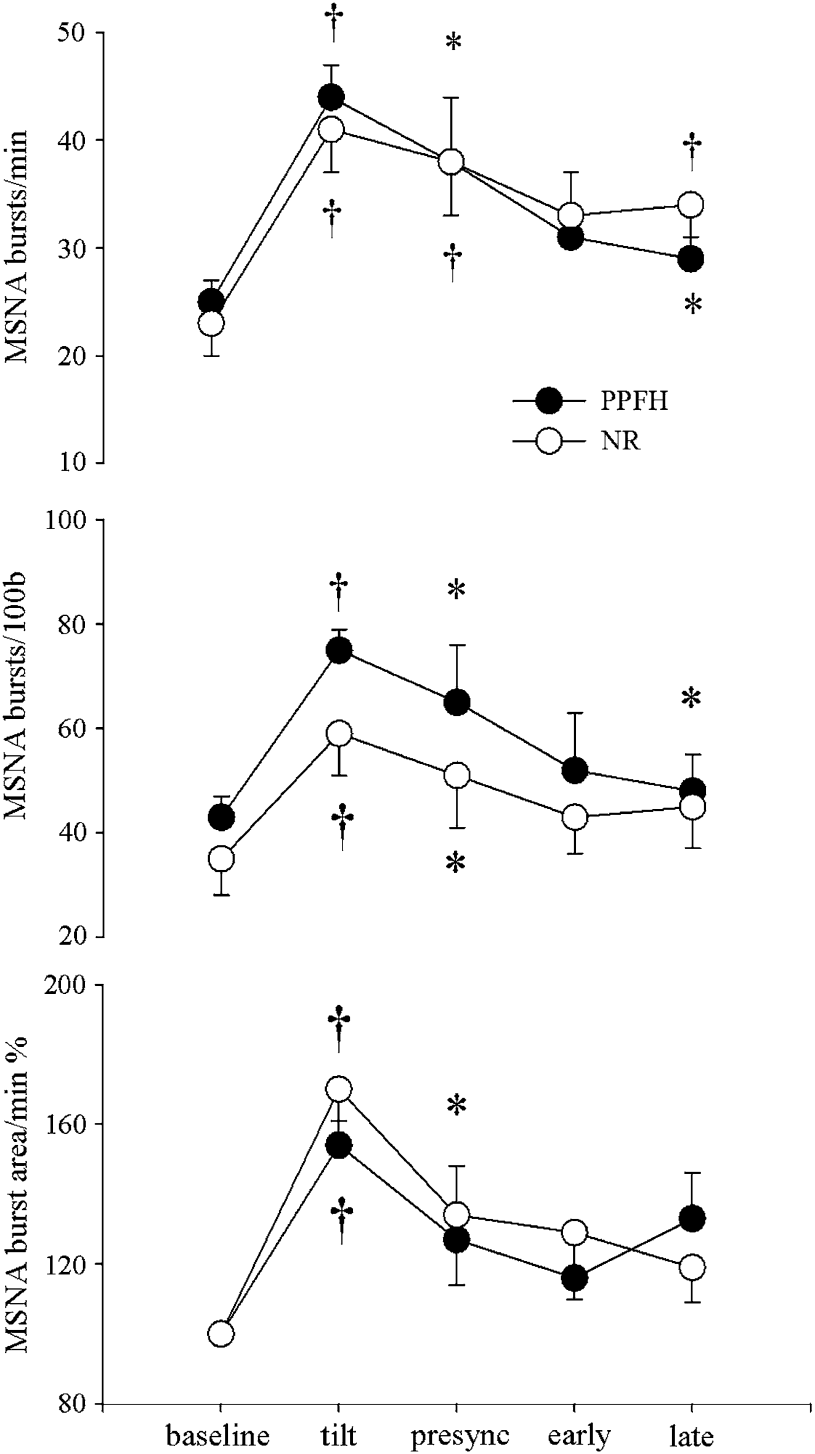
Mean and standard error for MSNA burst frequency, burst incidence and burst area at baseline, early tilt, presyncope, early and late recovery in patients with prolonged post-faint hypotension (PPFH *filled circles*) and patients with normal recovery (NR *open circles*). *Asterisks* and *dagger* refer to *p* < 0.05 and *p* < 0.01 for within-group comparisons to baseline

Between group comparisons of haemodynamic parameters across all time points showed that MAP (*p* = 0.004), and HR (*p* = 0.023) were lower in the in the PPFH group whereas SV was higher (*p* = 0.037). For all other variables, including CO (*p* = 0.89) and TPR (*p* = 0.128) and indices of MSNA (bursts/min *p* = 0.77, bursts/100b *p* = 0.65, and burst area/min *p* = 0.78) there were no significant differences between groups.

### Baseline

Between groups, the PPFH group had lower baseline BP and TPR than the NR group: MAP 84 ± 4 versus 104 ± 5 mmHg (*p* = 0.012); TPR 0.92 ± 0.1 versus 1.4 ± 0.3MU (*p* = 0.049), whereas SV was higher: 103 ± 10 versus 77 ± 4 ml (*p* = 0.046). HR, CO and all indices of MSNA were similar.

### Early HUT

In the PPFH group MAP and HR increased to 92 ± 5 mmHg (*p* = 0.043) and 66 ± 2 bpm (*p* = 0.006) respectively, while SV and CO decreased to 75 ml and 4.9 l/min (*p* = 0.004, *p* = 0.004). TPR tended to increase from 0.92 ± 0.1 to 1.2 ± 0.1MU (*p* = 0.15). In the NR group, MAP remained constant at 108 ± 5 mmHg (*p* = 0.2), while HR increased to 84 ± 6 bpm (0.02). SV and CO decreased to 54 ± 6 ml (*p* = 0.004) and 4.3 ± 0.4 l/min (*p* = 0.02) while TPR tended to increase (*p* = 0.1). MSNA indices increased in both groups: PPFH 25 ± 2 to 44 ± 3 bursts/min (*p* = 0.001), 43 ± 4 to 75 ± 4 bursts/100beats (*p* = 0.001) and 100 to 154 ± 7% burst area (*p* = 0.001); NR 23 ± 3 to 41 ± 4 bursts/min (*p* = 0.001), 35 ± 7 to 59 ± 8 bursts/100beats (*p* = 0.001) and 100 to 170 ± 13% burst area (*p* = 0.001).

### Presyncope (last minute before tilt back)

MAP fell below baseline levels in both groups: PPFH 68 ± 7 (*p* = 0.045) and NR 70 ± 7 mmHg (*p* = 0.002). In the PPFH group HR increased to 75 ± 6 bpm (*p* = 0.027), SV fell to 54 ml (*p* = 0.003), and CO to 4.0 l/min (*p* = 0.002). In the NR group, SV fell further from baseline to 51 ml (*p* = 0.012). MSNA indices began to fall but remained above baseline levels in both groups: PPFH 38 ± 5 bursts/min (*p* = 0.03), 65 ± 11 bursts/100 beats (*p* = 0.04), and 149 ± 21% burst area (*p* = 0.06); NR 38 ± 5 bursts/min (*p* = 0.01), 51 ± 10 bursts/100 beats (*p* = 0.03) and 157 ± 20% burst area (*p* = 0.02).

### Early recovery

In both groups, MAP remained below baseline: PPFH 51 ± 5 (*p* = 0.001) and NR 83 ± 5 mmHg (*p* = 0.001). In the PPFH group, HR fell to baseline at 56 bpm (*p* = 0.24), while SV and CO remained below baseline at 69 ± 6 ml (*p* = 0.009) and 3.7 ± 0.5 l/min (*p* = 0.001). In the NR group SV began to increase but remained below baseline at 62 ml (*p* = 0.049). All MSNA indices returned to baseline levels in both groups. PPFH 31 ± 6 bursts/min (*p* = 0.09), 52 ± 11 bursts/100 beats (*p* = 0.11) and 133 ± 13% burst area (*p* = 0.16); NR 33 ± 3 bursts/min (*p* = 0.06), 43 ± 7 bursts/100 beats (*p* = 0.25), 129 ± 19% burst area (*p* = 0.16).

### Late recovery

MAP remained below baseline in both groups: 64 ± 5 and 93 ± 5 mmHg, respectively (*p* = 0.001, *p* = 0.03). In the PPFH group, SV recovered but CO remained below baseline at 88 ± 6 ml (*p* = 0.178) and 4.6 l/min (*p* = 0.049), respectively. In the NR group, SV increased back to baseline: 65 ml (*p* = 0.127). MSNA indices remained at or above baseline levels in both groups. PPFH 29 ± 4 bursts/min (*p* = 0.02), 48 ± 7 bursts/100 beats (*p* = 0.05), 133 ± 13% burst area (*p* = 0.08) and NR 34 ± 3 bursts/min (*p* = 0.01), 45 ± 8 bursts/100 beats (*p* = 0.09), 119 ± 10% burst area (*p* = 0.09).

In summary, MAP and HR were lower at baseline and during recovery in the PPFH group. We also observed that unlike the NR group, CO was depressed through recovery in the PPFH group. Although TPR was lower in the PPFH group at baseline, this was not apparent during recovery and exaggerated MSNA withdrawal was not seen.

## Discussion

Our hypothesis, that MSNA would remain increased from baseline values during PPFH, is supported by our findings. TPR remained constant at baseline levels in both groups, while MSNA tended to be elevated. Thus although blood pressure was clearly lower in the PPFH group during recovery, it did not appear to be mediated by an exaggerated withdrawal of MSNA. Delayed recovery of CO and a lower HR throughout recovery secondary to increased vagal outflow is therefore the most likely mechanism for PPFH. These results are consistent with the findings of Wieling et al. [19] who first described PPFH in a group of seven patients during HUT. Wieling concluded that decreased LV contractility, secondary to exaggerated vagal activity was responsible for PPFH. Like his patients, ours were predominantly middle-aged males, with low-normal resting BP, some of whom had blood phobia. Unfortunately, our methods for deriving SV, CO and TPR did not allow reliable comparisons of baseline levels so we are uncertain as to the hemodynamic mechanism for the low-normal resting BP in the PPFH group. Although MSNA is an important determinant of TPR in the dynamic setting [10], the relationship between MSNA and TPR at rest is more complicated, particularly with increasing age [27]. We measured similar MSNA levels in the two groups at baseline and throughout tilt, therefore, we think TPR was unlikely to be lower in the PPFH group. Therefore, our data do not support the hypothesis that PPFH patients have low resting TPR, nor impaired sympatho-vasoconstriction predisposing them to VVS [8, 28].

Even though MSNA has been studied extensively during tilt and presyncope [5–8, 28, 30], few studies include MSNA during recovery [13, 30]. This is because it is technically very difficult to maintain a satisfactory nerve recording field during the course of presyncope and recovery. The recording electrode may be dislodged by muscle tensing, myoclonic leg movements or the tilt-back maneuver. In some studies, the resulting decrease in burst frequency or size may have been falsely interpreted as sympathetic withdrawal [31]. The only way to be sure that a field has been maintained throughout presyncope is to observe spontaneous recovery of the bursting pattern after tilt-back without adjusting the recording electrode [8]. Although MSNA remained well above baseline during presyncope, we observed a terminal fall-off in MSNA immediately before tilt-back in both groups. This data confirms previous studies showing that provided patients became sufficiently hypotensive, some degree of sympathetic withdrawal usually occurred during the final seconds of presyncope before tilt-back [5, 8, 13, 28]. This is partly because individual bursts of MSNA are gated to the diastolic phase of the cardiac cycle and when heart rate decreases, burst rate follows [32]. Two recent studies have contested this finding [29, 31].We observed that the decrease in MSNA burst rate was not totally dependent on bradycardia because burst incidence usually decreased in unison with burst rate. For ethical reasons, and to decrease the risk of losing the recording field, patients were tilted back to the horizontal before bradycardia and complete sympathetic withdrawal ensued. Consequently, we demonstrated only partial sympathetic withdrawal at tilt-back and thereafter, maintenance of MSNA at or above baseline levels during recovery. We are uncertain as to why MSNA was not higher in the PPFH group at this time and cannot exclude some degree of central sympathetic depression. Nevertheless, our data demonstrated delayed recovery of CO and lower HR in the PPFH group, which implies that this is the most likely mechanism responsible. Therefore, doctors with a specialist interest in syncope should be aware that a minority of fainters (7.4% in this study and 1.9% in the study by Wieling et al. [19] may exhibit PPFH, for example during tilt-testing and so will require monitoring and rest in the horizontal position for an extended recovery period. This phenomenon seems to be secondary to a transient vagal-induced paresis of the left ventricle [19], which is a fascinating and novel physiological prospect. We predict that passive leg raising, a manoeuvre that rapidly increases venous return, will not decrease recovery time during PPFH. A central stimulation by “dynamic tension” (active bilateral leg flexion and extension) may be required [19] (Wieling et al. 2012 Case).

### Limitations

This is a retrospective study, looking at a relatively small group of patients, which was limited mainly by technical difficulties obtaining satisfactory MSNA data. At the time the recordings were made, we were mainly concerned with preserving the recording field throughout presyncope, and so we did not allow our patients to become as bradycardic as the PPFH group previously reported [19]. This accounts for the difference in hemodynamics between the two studies. Nevertheless, this should not detract from our main finding, that MSNA is not decreased during PPFH. MSNA recordings, although the best dynamic measure of sympatho-vasoconstriction, are all limited to the vascular bed of one (lower) limb, which may not be representative of the major resistance/capacitance vessels during vasovagal syncope [33]. We did not measure lower limb blood flow and so cannot exclude loss of sympathetic transduction or active vasodilatation as a possible mechanism for increased MSNA during PPFH [31].
